# Supra-inguinal fascia iliaca block under ultrasound guidance for perioperative analgesia during bipolar hip arthroplasty in a patient with severe cardiovascular compromise: A case report: Retraction

**DOI:** 10.1097/MD.0000000000020195

**Published:** 2020-04-24

The article, “Supra-inguinal fascia iliaca block under ultrasound guidance for perioperative analgesia during bipolar hip arthroplasty in a patient with severe cardiovascular compromise: A case report”,^[1]^ is being retracted by request of the authors due to self-reported misconduct. In the preoperative evaluation of the patient, the article incorrectly reported that the patient had severe heart failure with an ejection fraction of 33%. The patient did not have severe heart failure. The management method in perioperative time was incorrectly reported as of dexmedetomidine infusion. The patient was managed by propofol and remifentanil infusion.
